# Surface fungal diversity and several mycotoxin-related genes’ expression profiles during the Lunar Palace 365 experiment

**DOI:** 10.1186/s40168-022-01350-8

**Published:** 2022-10-12

**Authors:** Jianlou Yang, Zikai Hao, Lantao Zhang, Yuming Fu, Hong Liu

**Affiliations:** 1 Key Laboratory for Biomechanics and Mechanobiology of the Ministry of Education, Beijing Advanced Innovation Centre for Biomedical Engineering, School of Biological Science and Medical Engineering, Beihang University, Beijing, 100191 China; 2 China Academy of Space Technology, Beijing, 100094 China; 3 International Joint Research Center of Aerospace Biotechnology & Medical Engineering, Beihang University, Beijing, 100191 China; 4 State Key Laboratory of Virtual Reality Technology and Systems, School of Computer Science and Engineering, Beihang University, Beijing, 100083 China

**Keywords:** Lunar Palace 1, Lunar Palace 365 experiment, Fungal community, Mycotoxin genes

## Abstract

**Background:**

Chinese Lunar Palace 1 (LP1) is a ground-based bio-regenerative life support system (BLSS) test bed integrating highly efficient plant cultivation, animal protein production, urine nitrogen recycling, and bioconversion of solid waste. To date, there has been no molecular method-based detailed investigation of the fungal community and mycotoxin potential in BLSS habitats. To ensure safe BLSS design for actual space missions, we analyzed the LP1 surface mycobiome and mycotoxin potential during the Lunar Palace 365 project through internal transcribed spacer region 1 (ITS1) amplicon sequencing and quantitative polymerase chain reaction (qPCR) with primers specific for *idh*, *ver1*, *nor1*, *tri5*, and *ITS1*.

**Results:**

The LP1 system exhibited significant differences in fungal community diversity compared to other confined habitats, with higher fungal alpha diversity and different community structures. Significant differences existed in the surface fungal communities of the LP1 habitat due to the presence of different occupant groups. However, there was no significant difference between fungal communities in the plant cabin with various occupants. Source tracker analysis shows that most of the surface fungi in LP1 originated from plants. Regardless of differences in occupants or location, there were no significant differences in mycotoxin gene copy number.

**Conclusions:**

Our study reveals that plants are the most crucial source of the surface fungal microbiome; however, occupant turnover can induce significant perturbations in the surface fungal community in a BLSS. Growing plants reduced fungal fluctuations, maintaining a healthy balance in the surface fungal microbiome and mycotoxin potential. Moreover, our study provides data important to (i) future risk considerations in crewed space missions with long-term residency, (ii) an optimized design and planning of a space mission that incorporates crew shifts and plant growth, and (iii) the expansion of our knowledge of indoor fungal communities with plant growth, which is essential to maintain safe working and living environments.

Video Abstract

**Supplementary Information:**

The online version contains supplementary material available at 10.1186/s40168-022-01350-8.

## Background

Future, manned deep-space exploration, including the construction and utilization of artificial lunar bases and the human exploration of Mars [1], will require the construction of safe, confined habitats, a central component of which is a bioregenerative life support system (BLSS) [2, 3]. A BLSS is a small, balanced, self-sufficient artificial ecosystem that encloses air, food, and water loops for crews in confined isolation environments [4, 5]. Bio-contamination has long been a subject of great interest in building construction, especially in confined and closed environments such as a BLSS. Mycotoxins have a more important impact on human health than toxic bacterial metabolites, so they have received more rigorous attention in indoor analytical assessments [6, 7]. Several studies have shown that indoor fungal exposure is associated with numerous health problems, such as dermal symptoms and sick building syndrome [8, 9]. An important cause of these phenomena is the mycotoxins that a range of fungi can produce; these active compounds linger in indoor or confined environments, even after the fungal cells are no longer viable [10]. Mycotoxins are significant contaminants that can cause acute and chronic toxic effects in humans at very low concentrations (ppb to ppt) [11]. Due to the multitude of mycotoxins in indoor environments, most of the analytical methods applied so far were developed to specifically assess the presence of a restricted set of mycotoxins of primary toxicological interest [12–15]. Therefore, the present study also considered the potential of mycotoxin contamination of the confined environment.

Recent investigations have examined the fungal microbiome in authentic physicochemical regenerative life support systems (such as the International Space Station, ISS) [16] and ground analogs (such as the inflatable lunar/Mars analog habitat, ILMAH) [17], as well as in the controlled environment of a spacecraft assembly and integration test center (AIT) associated with the ISS. These previous studies have implicated surfaces as major contamination routes by which occupants may come into contact with fungi and mycotoxins [18]. The building design frameworks of BLSSs are distinct from physicochemical regenerative life support systems (PCSSs) by the BLSSs’ biological components, particularly the intensive cultivation of plants. It is now known that building design patterns strongly affect fungal communities in fabricated environments [19]. Therefore, it is essential to characterize the fungal microbiome and mycotoxin potential in analogous habitats to ensure safe BLSS designs for future space missions. Thus far, there have been no detailed reports of the fungal community and mycotoxin genes in habitats analogous to BLSSs.

Chinese Lunar Palace 1 (LP1) was established as a ground-based BLSS test bed, integrating highly efficient plant cultivation, animal protein production, urine nitrogen recycling, and bioconversion of solid waste [5]. LP1 was utilized to examine and overcome the various technical and scientific challenges in creating a closed and isolated extraterrestrial living space. In the present study, we comprehensively characterized the fungal communities and mycotoxin-producing genes in a long-term BLSS analog experiment named “Lunar Palace 365” in LP1. Using qPCR and amplicon sequencing of the nuclear ribosomal internal transcribed spacer (ITS) region, we explored the fungal communities and mycotoxin genes on LP1 surfaces from different defined locations over two crew groups, spanning 370 days. In addition, the fungal microbiome data in LP1 were compared with that in other PCSSs, including the ISS [16], ILMAH [17], and an AIT center [20].

We hypothesized that (i) the LP1 system exhibits significant uniqueness in fungal community diversity compared to other confined habitats, (ii) plants can mitigate human-induced fluctuations in fungal communities and maintain the fungal diversity since plants are the most important contributor to the surface fungal community, and (iii) the fungal community does not harbor a large number of accumulated toxin gene copies because of the green ecological functions within the BLSS. The results of our study provide new insights into the mycobiome of the closed, man-made ecological systems and may facilitate the development of fungal contamination control strategies for maintaining BLSS occupant health, thus furthering our progress towards human habitation in deep space.

## Methods

### The Lunar Palace 1 habitat and Lunar Palace 365 project

**Fig. 1:**
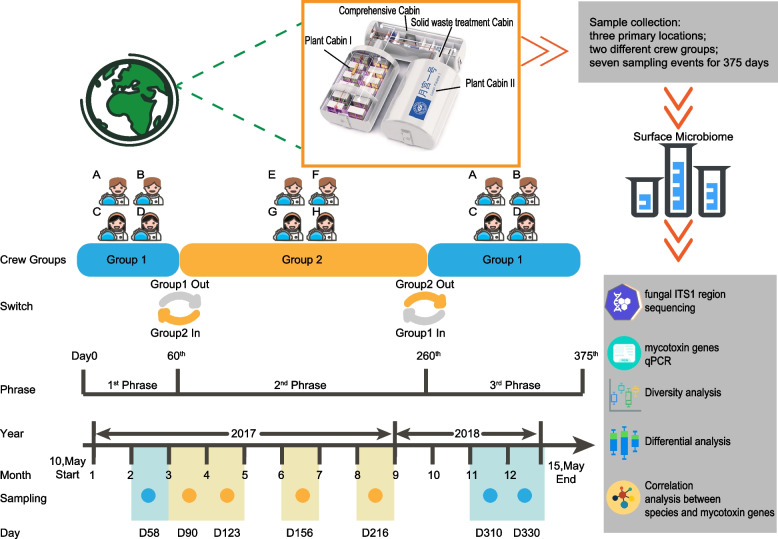
Overview of the experimental design and sample collection (surface microbiome) in LP1

In brief, LP1 is a BLSS test bed located in Haidian, Beijing, China (116° 25′ 29″ E, 39° 54′ 20″ N), which occupies a total area of 160 m^2^ and has a total volume of 500 m^3^. LP1 has two plant cabins (PC, each 10 × 6 × 3.5 m^3^) and one comprehensive cabin (CC, 14 × 3 × 2.5 m^3^) which contains 4 private bedrooms, a living room, a bathroom, an insect culturing room, and a solid waste treatment cabin (SC, 3.2 × 2.3 × 3.5 m^3^) [21, 22] (Fig. 1).

The research we describe here was part of the Lunar Palace 365 project carried out in the LP1. The Lunar Palace 365 project was launched on May 10, 2017, by the Institute of Environmental Biology and Life Support Technology, Beijing University of Aeronautics and Astronautics. A total of eight volunteers were divided into two groups (G1 and G2; 2 females and 2 males each) and spent a total of 370 days in the LP1. The project was divided into three phases: the first phase lasted for 60 days with the four crew members of G1 (May 10 to July 10, 2017), the second phase lasted for 200 days with the four crew members of G2 (July 10, 2017, to January 26, 2018), and the third phase lasted for 110 days with the four crew members of G1 (January 26 to May 15, 2018). Surface samples were collected during seven sampling events (day 58 [D58], day 90 [D90], day 123 [D123], day 156 [D156], day 216 [D216], day 310 [D310], and day 330 [D330]) from three primary locations (CC: comprehensive cabin; PC: plant cabin; SC: solid waste treatment cabin; Fig. 1). Prior to inhabitation, the LP1 surfaces were cleaned according to established protocols; during the experimental period, the surfaces were cleaned regularly.

### Sample collection and processing

Surface samples were collected by the same occupant in each group at various time points and locations throughout the mission. A sterile swab tube (0.85% NaCl, 3 mL, Jiangsu Rongye Technology Co., Ltd.) was used at the sampling point using a 10 cm × 10 cm standard sterilization specification plate. After sampling, the occupant immediately sealed and labelled the sampling tube and transported it out of the system through a small logistics channel for experiments. Then, the external experimenters quickly performed the subsequent DNA isolation. The samples were centrifuged at 1400 ×*g* for 5 min to separate the fluid from the swab. The separated fluid was subjected to DNA extraction and stored at −80 °C, at which time the swab was discarded.

Appropriate field controls were taken by waving the sampling tool (swab) through the air at the LP1 facility for a few seconds, representing the so-called field blanks. This procedure was performed at least once per sampling event. Unused sampling material was processed along with the samples and served as lab controls.

### DNA extraction

Samples were thawed at 4 °C overnight before transferring them to DNA-free centrifuge tubes filled with polymerase chain reaction (PCR)-grade water. The fluid was transferred to 2.0 ml centrifuge tubes. The sample tubes were then placed in a shaking mixer and incubated at 70 °C for 10 min at 1000–1200 rpm to promote sample lysis. FastDNA Spin Kits (MP Biomedicals (Beijing) Co., Ltd. Beijing, China) were used to isolate the DNA according to the manufacturer’s instructions.

### Molecular fungal community analysis using Illumina sequencing

A two-step amplification process was applied prior to MiSeq Illumina sequencing. In the first step, the extracted DNA was used as the template to design primers with connectors for PCR; in the second step, PCR was performed using the PCR products from the first step as the templates: the forward primer ITS1F sequence was 5′-CTTGGTCATTTAGAGGAAGTAA-3′, and the reverse primer ITS2R sequence was 5′-GCTGCGTTCTTCATCGATGC-3′. Amplification was performed in 10 μL reactions with 50 ng ± 20% genomic DNA, 0.3 μL each 10 μM primers, 5 μL KOD FX Neo Buffer (Toyobo [Shanghai] Biotech Co., Ltd.), 2 μL 2 mM dNTP, 0.2 μL KOD FX Neo (Toyobo [Shanghai) Biotech Co., Ltd.), and ddH_2_O added to 10 μL. The reactions were performed on a 96-well PCR system (Applied Biosystems, AB, 9902) under the following thermal profile: 95 °C for 5 min (initial denaturation) and then 25 cycles of 95 °C for 30 s, 50 °C for 30 s, and 72 °C for 40 s, followed by one cycle of 72 °C for 10 min and a 4 °C hold. The PCR products were collected and resolved on a 1.8% agarose gel, purified using the MinElute PCR purification kit according to the manufacturer’s instructions, and quantified using a QuantiFluorTM-ST (Promega [Beijing] Biotech Co., Ltd., Beijing, China). The amplified target fragment was incubated with bridge PCR compatible primers and further amplified by Solexa PCR [23]. After 1.8% agarose gel electrophoresis at 120 V for 40 min, the target fragment was excised and sequenced on an Illumina MiSeq platform at Biomarker Technologies Co., Ltd. (Beijing, China). Negative controls, including no template (three replicates) and template from unused swabs (three replicates), were also subjected to amplification. Consequently, fungal ITS sequences of these negative controls could not be amplified, indicating that both PCR reagents and sample DNA were not contaminated.

### qPCR (ITS region and mycotoxin genes)

The overall microbial load of surface samples was determined by real-time quantitative polymerase chain reaction (qPCR) of the fungal ITS region. Fungus-specific primers targeting the ITS regions NS91 and ITS51 (primer pair NS91-ITS51 [24] for fungi, Additional file 1: Supplementary Table S1) were used for this analysis. qPCR assays targeting mycotoxin-producing fungal genes were performed using fluorescence-based detection in a Roche 13200 PCR device (Roche Diagnostics GmbH). Primer sequences for the mycotoxin genes are listed in Additional file 1: Supplementary Table S1 and have been described previously [25, 26]. Mycotoxin gene DNA standards generated from pure plasmids (Puc57) ranging from 10^0^ to 10^6^ were included in all reactions. The PCR amplification program was as follows: 95 °C for 10 min (initial denaturation), 40 cycles of 95 °C for 15 s, and annealing at 59 °C for 45 s using a Hybaid, Omn-E Thermal Cycler. The total reaction mixture (20 μL) comprised 2 × SYBR Green qPCR Master Mix (10 μL), forward primer (1 μL), downstream primer (1 μL), DNA template (1 μg), and ddH_2_O to 20 μL.

### ITS raw data processing and quality control

The unambiguous DNA sequences were subjected to the QIIME2 (2020.02 version) pipeline [27]. Briefly, reads were demultiplexed using the QIIME2 demux plugin (*qiime demux summarize*; summarize counts per sample for all samples and generate interactive positional quality plots based on “n” randomly selected sequences.) according to their barcode sequence. Demultiplexed sequences were further quality filtered and clustered using the DADA2 (https://benjjneb.github.io/dada2/index.html) plugin (*qiime dada2 denoise-paired*; this method denoises paired-end sequences, dereplicates them, and filters chimeras.), and reads were truncated to avoid low-quality scores (> 250 bp for forward, > 250 bp for reverse reads; trim-left-f = 22, trim-left-r = 20, trunc-q = 5). The QIIME2 DADA2 was used for quality control and processing (including chimera removal) of the raw demultiplexed reads resulting in an amplicon sequence variant (ASV) table, a “higher resolution analogue of the traditional operational taxonomic unit (OTU) table” [28].

The taxonomic analysis was carried out with the QIIME2 feature-classifier plugin; taxonomy was assigned to ASVs with the UNITE dynamic database [29] (99% similarity, version 8.3, release date 2021-05-10; *qiime feature-classifier classify-sklearn*; classify reads by taxon using a fitted classifier.). We used the QIIME2 ANOCM plugin (*qiime composition ancom*; apply analysis of composition of microbiomes [ANCOM] to identify features that are differentially abundant across groups) to analyze the abundance of differences between groups. To generate trees for phylogenetic diversity analysis (including Faith’s phylogenetic diversity, weighted UniFrac, unweighted UniFrac, and phylogenetic trees), we used the QIIME2 phylogenetic plugin (*qiime phylogeny*; this QIIME 2 plugin supports generating and manipulating phylogenetic trees.) to perform multiple sequence alignment, remove regions of high variability, build trees, and convert unrooted trees to rooted trees. We then calculated alpha diversity indices (richness, Shannon index, and Chao1; *qiime diversity core-metrics*; applies a collection of diversity metrics [non-phylogenetic] to a feature table) and beta diversity metrics (Bray-Curtis, Jaccard, weighted UniFrac, and unweighted UniFrac) through the diversity plugin.

### Bioinformatics and statistical analysis

To assess the differences in fungal diversity between the different crew groups, the following univariate statistical analyses were carried out using specific packages in R (v4.0.2; http://www.R-project.org/), unless otherwise noted. The normal distribution of the datasets was tested using the Shapiro-Wilk normality test, and, as most were not normally distributed (*p* < 0.05), we used a Wilcoxon rank-sum test to investigate the differences. Beta diversity comparisons were performed using unweighted UniFrac. Dimensionality reduction on the UniFrac distances was performed through principal coordinates analysis (PCoA). We used PERMANOVA [30] (permutational multivariate analysis of variance, with 999 permutations) to test whether the sample groups harbored significant differences in the microbial community structure in the PCoA. Resulting *p*-values were corrected using the Benjamini-Hochberg correction. Alpha diversity metrics (Shannon’s diversity, richness) and beta diversity metrics were calculated in QIIME2 [27]. Visualizations were conducted using MicrobiotaProcess packages [31] in R. Statistical significance was defined as *p* < 0.05.

The taxonomy data used in various analyses were summarized from the ASV data using QIIME2. For bar plots, the data were normalized by total sum normalization. The taxonomic composition of each group was visualized as a stacked bar plot at the phylum and genus levels using the ggplot2 package. EdgeR [32] (*p* < 0.05, *FDR* < 0.2) was also used to identify significant differences in the relative abundances of different taxa between the groups. Significance was based on the Benjamini-Hochberg corrected *p*-value of the Wilcoxon rank test (significance threshold *p* < 0.05).

To identify possible biomarkers between G1 and G2, we performed linear discriminant analysis (LDA) effect size (LEfSe) method (http://huttenhower.sph.harvard.edu/lefse/) based on the ASVs (the *p*-value of Kruskal-Wallis = 0.05, LDA score = 4.0) [33]. To compare the proportion of shared and exclusive markers of different taxa between the different closed habitats, Venn diagrams were also generated using the ggvenn package [34]. A co-occurrence network of the surface samples was generated using the Spearman correlation matrix constructed with Gephi [35] to assess the complexity of the microbiota interactions.

To obtain the best discriminant output of taxa across different locations in the LP1, we regressed the relative abundances of fungal taxa at the genus level against different sites in the LP1 using the default parameters of the R implementation of the algorithm (R package randomForest, ntree = 1000, default mtry of *p*/3, where *p* is the number of taxa per genus). In over 100 iterations, randomForest created lists of taxa ranked in order of feature significance. With five repeats, the number of marker taxa was determined using a tenfold cross-validation implemented with the rfcv() feature in the randomForest module. When using 23 essential classes, the number of genera against the cross-validation error curve stabilized. Heatmaps of the rho values were generated using the heatmap.2 function in the gplots package. Spearman’s rho values and corresponding *p*-values for the correlation analyses between phyla (clr-transformed data) from samples and mycotoxin genes were generated in R using the rcorr function.

Differences between sample types within the constrained environment were predicted using the SourceTracker algorithm [36]. Surface fungal community source tracing was performed in QIIME after closed-reference and open-reference ASV pickup using the LP1 compartment surface samples as a sink and plant compartment samples [37], human skin [38], human gut fungi [39], human oral fungi [40], and indoor air fungi [41] as alternative sources.

Furthermore, to investigate the function of the fungal community, the FUNGuild [42] and FungalTraits [43] tools were used to taxonomically parse fungal ASVs into several ecological categories [44].

### Comparison of the LP1 environmental microbiome with the other confined environment microbiomes

The published ISS project [16] amplicon sequence variant (ASV) table generated from fungal ITS region iTag sequencing was obtained from https://static-content.springer.com/esm/art%3A10.1186%2Fs40168-019-0666-x/MediaObjects/40168_2019_666_MOESM2_ESM.xlsx. ASV tables from the ILMAH microbiome study [17] were obtained from https://static-content.springer.com/esm/art%3A10.1186%2Fs40168-017-0280-8/MediaObjects/40168_2017_280_MOESM12_ESM.zip. Fungal ASV tables of the facilities used to assemble, test, and launch the OSIRIS-REx spacecraft (assembly, integration, and test; AIT) [20] were obtained from https://www.frontiersin.org/articles/10.3389/fmicb.2020.530661/full#supplementary-material. We merged the ASV tables and rarefied to the lowest number of reads recovered from all samples (9150 reads) in R and calculated the Bray-Curtis distance using the vegan package [45]. The normal distribution of the datasets was tested using the Shapiro-Wilk normality test; most were not normally distributed (*p* < 0.05). Alpha diversity analysis was carried out using the vegan package (v2. 5–6) in R v4.0.2. Differences in Shannon’s index, the Chao1 index, Pielou index, and Simpson index between groups were evaluated using the Kruskal-Wallis test. We used PCoA to compare the *β*-diversity based on Bray-Curtis distances of fungal communities in different environments and then used PERMANOVA to test whether the sample groups harbor significant differences in microbial community structure in the PCoA. For bar plots, the data were normalized by total sum normalization. The taxonomic composition of each group was visualized as a stacked bar plot at the phylum and genus levels using the ggplot2 package.

We performed SVM (support vector machine) analysis to predict the confined environment based on the ASV characteristics of the sample. Next, we randomly divided the data into two-halves (training set and test set), performed a fivefold cross-validation on the training set to adjust the SVM, and then analyzed the prediction error rate on the test set, using linear kernel prediction. These methods were implemented using scikit-learn, run SVM, and random forest machine analysis [46].

## Results

### Sequencing analysis and data processing

A total of 4,439,726 raw sequences were generated. Of these, 3,458,067 high-quality reads were retained after denoising and removing low-quality sequences and chimeric sequences with DADA2, subsequently generating 3468 ASVs (Additional file 1: Supplementary Table S2). Among them, 1055 ASVs, accounting for 2.50% (86,480 sequences) of the total high-quality sequences, could not be identified to any known phylum based on the UNITE database (99% similarity; version 8.3; release date 2021-05-10). Illumina-based reads of the lab and field controls showed negligible signals from DNA contamination and hence were not included in the subsequent analyses.

### Comparison of LP1 environmental microbiome with other confined environment microbiomes

**Fig. 2:**
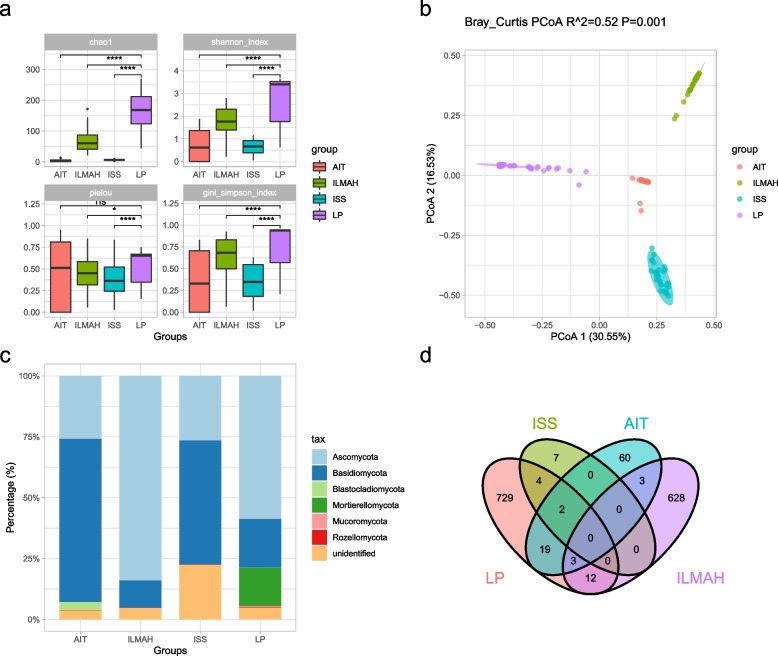
General characterization of the fungal microbiome in LP1 and other confined environments. **a** Alpha diversity of fungal communities. **b** Beta diversity estimates of the fungal communities between different confined environments (PERMANOVA test [with 999 permutations], significance threshold, *p* < 0.05). **c** Relative abundance (%) of the major phyla present in the fungal microbial communities. **d** Venn diagram of the numbers of shared and unique ASVs observed in the fungal microbial communities. LP, Lunar Palace 1; ISS, International Space Station; AIT center, assembly, integration, and test center; ILMAH, inflatable lunar/Mars analog habitat

Publicly available sequences of samples collected from ISS dust, AIT surfaces, and surface samples from the ILMAH were compared with the LP1 environment fungal microbiome. The resulting merged fungal ASV table contains data from 163 individual surface samples of over 1467 taxa (Additional file 1: Supplementary Table S3). Next, we calculated the Chao1, Shannon, Pielou evenness, and Simpson indices. These analyses revealed an apparent higher fungal alpha diversity in LP1 than in the other confined environments (Fig. 2a). Moreover, as the PCoA plot shown in Fig. 2b indicates, the fungal microbiome on the LP1 surfaces is unique, differing from that in the ISS dust and AIT and ILMAH surfaces. Based on the Bray-Curtis distances, PC1 and PC2 were responsible for 30.61% and 16.53% of the total variation. Analysis by PERMANOVA tests showed significant differences from the control environments (*p* = 0.001).

At the phylum level, the confined environments harbored a higher relative abundance of Ascomycota and Basidiomycota; in particular, LP1 harbored higher relative abundances of Mortierellomycota and was dominated by the genera *Mortierella*, *Russula*, and *Trichoderma* (Fig. 2c; Supplementary Fig. S1). We also found 729 unique fungal ASVs in the LP1 environment (Fig. 2d).

With the SVM, we were able to predict the confined environments of origin of deidentified samples (i.e., those about which we knew the different controlled environments of origin but withheld that information from the classifier) with 100% accuracy, based only on the mycobiome composition (Supplementary Fig. S2; F-1 score, 1.0).

### Effects of crew members on the surface fungal microbiome

**Fig. 3:**
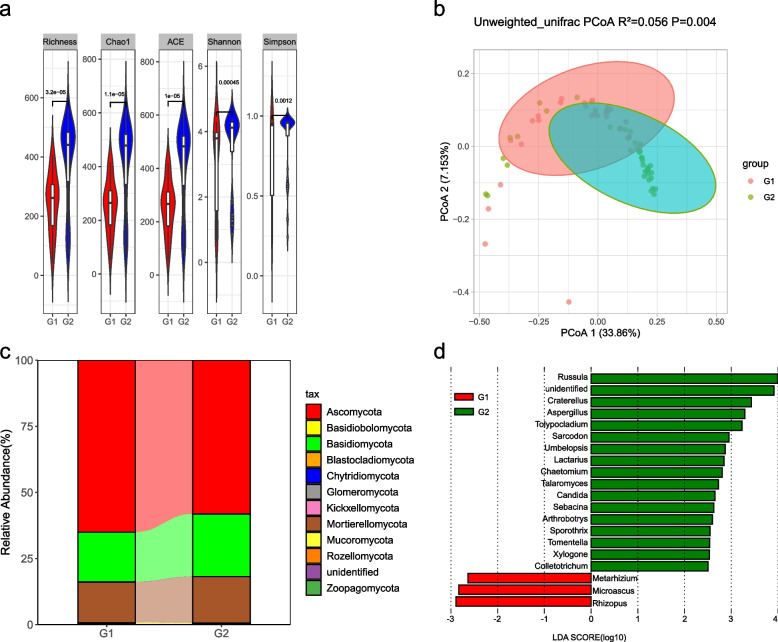
Fungal microbiome profile of LP1 environmental samples. **a** Alpha diversity estimates of the fungal communities (Wilcoxon rank test, significance threshold, *p* < 0.05). **b** PCoA performed at the ASV level for the ITS1 data set between the groups (unweighted UniFrac distance). G1, group 1 of crew members; G2, group 2 of crew members. **c** Relative sequence abundance of fungal phyla associated with G1 and G2. **d** LEfSe analysis identified the differentially abundant genera between G1 and G2 (LDA significance threshold > 2.5)

Fungal alpha diversity of the surface microbiome was comprehensively assessed using different indices (community richness [number of observed ASVs], Chao1, ACE, Shannon, and Simpson). All metrics revealed that G2 introduced a significantly greater fungal community diversity when compared with G1 (richness: *p* = 2.9e-05, Chao1: *p* = 1.1e-05, ACE: *p* = 1.1e-05, Shannon: *p* = 0.00045, Simpson: *p* = 0.0012; Fig. 3a).

We also compared the composition of fungal beta diversity within the different groups; PCoA was significantly dissimilar (*p* = 0.003, *p*-value evaluated via PERMANOVA) at the level of ASVs, based on the Jaccard distance. PCoA exhibited different degrees of clustering and different explanations (6.509–33.86%) of the total variation in the fungal communities between G1 and G2. Based on the unweighted UniFrac dissimilarity metrics, PC1 was responsible for 22.87% and PC2 for 6.509% of the total variation. The communities attributed to G1 were distinguishable from those of G2 with 90% confidence ellipses (Fig. 3b).

Eleven phyla (Ascomycota, Basidiomycota, Mortierellomycota, Mucoromycota, Rozellomycota, Chytridiomycota, Glomeromycota, Basidiobolomycota, Aphelidiomycota, Kickxellomycota, and Zoopagomycota) were classified using the UNITE reference database for all samples (the reads that were unclassified at the fungal phylum were removed from the sequencing data). As shown in Fig. 3c, the G1 fungi are mainly composed of Ascomycota (65.05%), while the G2 fungi are mainly composed of Ascomycota (58.15%) and Basidiomycota (23.66%) at the phylum level (Additional file 1: Supplementary Table S4).

The two groups of samples presented differences in taxonomic compositions at the genus level. *Penicillium* was widely present in G1 (12.56%). In contrast, its abundance in G2 (10.33%) was low. Furthermore, the percentage of *Mortierella* in G2 was 11.97%, while it was lower in G1 (4.90%; Fig. 3c, Additional file 1: Supplementary Table S5). At the genus level, we performed LEfSe analysis (Kruskal-Wallis; *p* < 0.05, LDA score > 2.5; Fig. 3d) to compare the significant differences in relative abundances between the groups. The amplicon sequence variants belonged primarily to nineteen genera: *Rhizopus*, *Microascus*, and *Metarhizium* were enriched in G1; *Russula*, *Craterellus*, *Aspergillus*, *Tolypocladium*, *Sarcodon*, *Umbelopsis*, *Lactarius*, *Chaetomium*, *Talaromyces*, *Candida*, *Sebacina*, *Arthrobotrys*, *Sporothrix*, *Tomentella*, *Xylogone*, and *Colletotrichum* were enriched in G2.

*Penicillium* was the most abundant organism in the surface samples in our dataset, which includes 90 ASVs that make up 2.6% of the total. Several species of *Penicillium* fungi are known mycotoxin producers, thus posing a potential threat to human health [14]. For this reason, we examined how the alpha diversity of the *Penicillium* sequences changed as a result of crew member presence. The G1 and G2 samples indeed show significant differences in richness (*p* = 0.00041; Supplementary Fig. S3a). As with the collective mycobiome, the Shannon’s diversity of *Penicillium* was lower in G1 samples compared to G2 (*p* = 0.00029; Supplementary Fig. S3b).

We were particularly interested in the change of the fungal community diversity over time, i.e., 370 days of confinement. When analyzing the fungal community diversity over time, we found that the diversity decreased and then increased and remained stable between the Shannon diversity index and days of isolation (Fig. S4). However, the community diversity was not strongly fluctuating over time (the Shannon diversity remained around four at most points in time). The ecological functions (trophic modes and guilds) in the groups (G1 and G2) are shown in Fig. S5 and Tables S2 and S6. The majority of the trophic modes fell into symbiotroph (13.76%), saprotroph (28.28%), and saprotroph-symbiotroph (15.51%) in G1. By contrast, G2 was mainly composed of saprotroph (18.13%), symbiotroph (17.24%), and saprotroph-symbiotroph (17.64%). Overall, we observed a decrease in saprotroph functions and an increase in symbiotroph functions from the microbiome in G1 to the microbiome in G2.

### Effects of sample location on the surface fungal microbiome

Samples from different sites within the same crew group (ANOSIM G1: *R* = −0.05, *p* = 0.88; G2: *R* = 0.004, *p* = 0.37; Supplementary Fig. S6 d and e) differed less than samples from the same site with a different crew group (ANOSIM CC: *R* = 0.098, *p* = 0.078; PC: *R* = 0.06, *p* = 0.11; SC: *R* = 0.1, *p* = 0.079; Supplementary Fig. S6 a–c); due to the different occupant composition, there was a significant difference in the fungal community on the surface of the equipment in the CC and SC, while there was no significant difference in the PC.

**Fig. 4:**
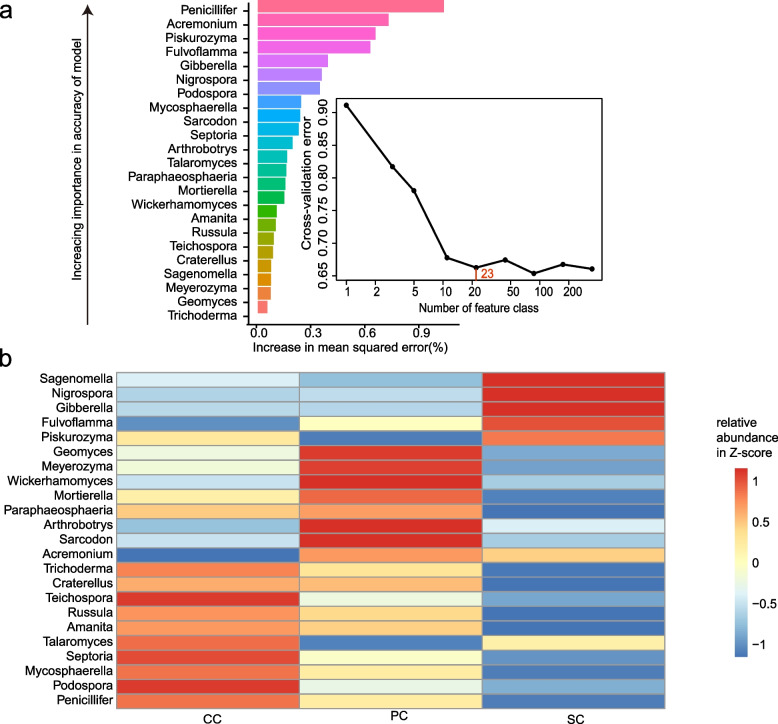
Fungal biomarkers associated with the different locations in LP1. **a** A random forest approach was used to identify 23 genera, associated with the indicated locations, ranked in order of contribution from largest to smallest. **b** Heatmap showing the relative abundance of the 23 location-related biomarkers. CC, comprehensive cabin; PC, plant cabin; SC, solid waste treatment cabin

To identify critical fungal classes as biomarker taxa to correlate with different locations in LP1, we performed a tenfold cross-validation with five repeats. The minimum cross-validation error was obtained when using 47 important classes. However, the number of classes against the cross-validation error curve stabilized when 23 classes were used (Fig. 4a). Thus, we defined these 23 classes as biomarker taxa in the model. The list of the 23 most abundant fungal taxa at the class level across the different locations in the LP1, in order of location-discriminatory importance, is provided in Fig. 4a. The majority of biomarker taxa, such as *Geomyces* and *Meyerozyma*, showed high relative abundance in the plant cabin (Fig. 4b).

The ecological functions (trophic modes and guilds) in the different locations (CC, PC, and SC) are shown in Fig. S7 and Tables S2 and S6. The majority of the trophic modes fell into symbiotroph (16.16%), saprotroph (18.82%), and saprotroph-symbiotroph (17.78%) in the CC. Similarly, the PC and SC were mainly composed of symbiotroph (14.98%, 16.36%), saprotroph (21.98%, 26.81%), and saprotroph-symbiotroph (16.67%, 15.75%). However, the proportion of trophic modes associated with pathotroph was low in the three locations.

### qPCR-based analysis of microbial quantity and surface mycotoxin genes

**Fig. 5:**
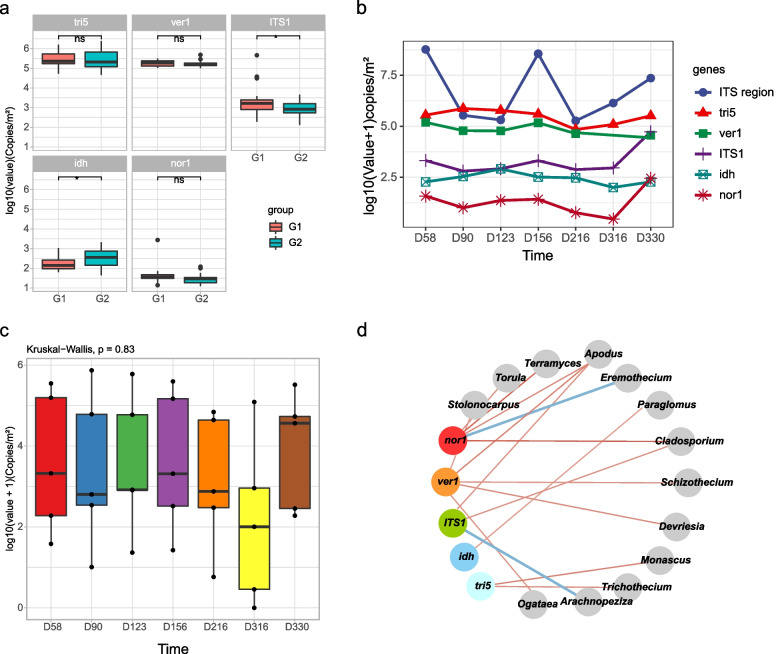
Changes, variations, and correlations of the fungal toxin genes. **a** Comparison of each mycotoxin gene copy number among the different occupant groups. **b** Changes in the mycotoxin gene copy number and microbial quantity over time. **c** Comparison of mycotoxin gene expression at different sampling times. **d** Correlation network analysis between mycotoxin genes and the surface mycobiome (*p* < 0.05, Spearman’s coefficient > 0.4). Each node represents taxa affiliated at the genus level (based on ITS1 rRNA), and the size of each node is proportional to the relative abundance of the genus. The lines between the nodes indicate positive connections among the genera. Each node is labelled at the module level

We quantitatively analyzed copy numbers of the ITS region for fungal load, as well as five representative genetic markers (*idh*, *ver1*, *nor1*, *tri5*, *ITS1*) of mycotoxin, including the aflatoxin, trichothecene, fumonisin, ochratoxin A (OTA), and patulin, as these mycotoxins are of the greatest significance to both human health and plant food security (Table S1). There was no significant difference in fungal toxin gene copy numbers either overall or between groups (Fig. 5a, Supplementary Fig. S8). Although fungal toxin gene copy numbers and fungal load (ITS region amplicons) were less volatile over time, there was no significant difference over the entire time series in general (Fig. 5b–c). Similarly, for the different locations, there was also no significant difference in fungal toxin gene copy numbers overall (Supplementary Fig. S9).

We examined the correlations between mycotoxin gene copy numbers and the relative abundances in the mycobiome. Spearman’s Rho correlation values between mycotoxin gene abundances and relative abundances of the surface mycobiome are shown in Fig. 5d. A strong positive correlation between fungi and mycotoxin genetic marker was observed. A summary of Spearman’s rho and *p*-values for all genera for each genetic marker is provided in Additional file 1: Supplementary Table S7. Furthermore, we conducted a co-occurrence network analysis to explore the complexity of connections within the fungal microbiome on the surfaces. We calculated the co-occurrence network’s topological characteristics and analyzed the Spearman correlation at the genus level. The fungal communities are generally positively correlated, and there is a strong mutualistic relationship (Supplementary Fig. S10).

### Source tracking of surface-related fungi within LP1

**Fig. 6:**
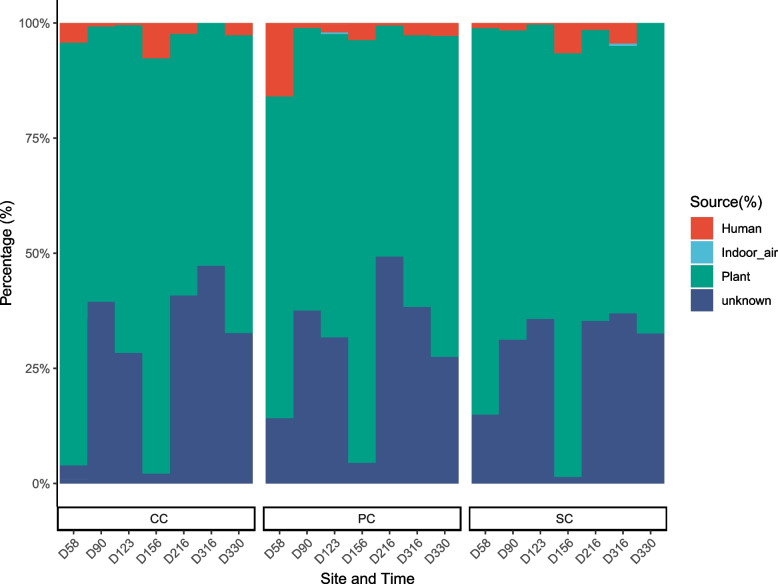
Source tracking of surface-related fungi within LP1, as measured using FEAST. CC, comprehensive cabin; PC, plant cabin; SC, solid waste treatment cabin

Finally, we assessed our data against publicly available datasets representing potential source environments. We found the following breakdown of surface fungal sources in LP1: plant, 68.75%; unknown, 28.12%; human, 3.08%; and indoor air, 0.05% (Fig. 6). Most fungal diversity correlated with plant fungal diversity and human skin fungal diversity. The former showed a larger proportion, suggesting a more significant impact on the LP1 surface fungal community profiles.

## Discussion

Exploring and understanding the fungal community dynamics of isolated and confined environments, including Earth-based space analogs and orbiting stations, facilitate the establishment and maintenance of safe life support systems for off-planet human habitation. Prior studies have noted the importance of studying fungal dynamics in real space life support systems [18]. Some fungal species have been shown to erode the integrity of the spacecraft itself through corrosion of metal parts and degradation of structural materials [47, 48]. Furthermore, some opportunistic fungal pathogens that may be able to infect crew members with compromised immune systems have been found on the ISS [16, 49]. Due to the logistical and funding constraints of studying space-faring vessels, fungal dynamics in ground-based analogs of crewed spaceflight, as an alternative, have served as proxies. For example, the mycobiome in the ILMAH has been examined [17]. However, the ground-based analogs explored thus far belong to the PCSS type of habitat. Bioregenerative life support systems (BLSSs), such as a lunar base or Mars base, are isolated, confined, and self-sufficient ecosystems that enclose air, food, and water loops and provide psychological benefits to long-duration space missions [4]. A BLSS, characterized by many biological components, particularly large plant-growing spaces, differs significantly from a PCSS. Furthermore, there have been few detailed investigations of microbial communities in ground-based BLSS analogs, especially with respect to fungal taxa. To fill this gap in our knowledge, we characterized the environmental mycobiome and mycotoxin genes from different locations of the LP1 ground-based BLSS test bed during two crew groups over an experimental period of 370 days. This enabled the examination of the temporal and spatial distribution of fungal populations in the LP1. We used qPCR and amplicon sequencing to generate a comprehensive surface fungal microbiome in the long-term confined and isolated environment containing plants, animals, and humans. Importantly, neither field nor lab controls showed amplifiable sequences, warranting no additional discussion.

The first question in our study was how the mycobiome in the LP1 ground-based BLSS test bed compares to other closed, regulated environments. We found that the LP1 system had a higher fungal alpha diversity and different community structures than other confined habitats (Fig. 2a and Fig. S2). Unlike Mars500, the community’s diversity did not fluctuate greatly over time. It is important to note that the community diversity recovered to a higher level and remained stable again after a brief decline due to the cleaning procedure. One explanation for the greater alpha diversity may involve a richer input of nutrients for fungi [50]. We speculate that large growing plants in a BLSSs provide a more abundant nutrient source for different fungi and maintain a diverse balance in the surface fungal microbiome. This may also be a reason for the numerous, unique fungal ASVs in LP1 (Fig. 2d). Consistent with previous reports [51, 52], the fungal majority in LP1 belonged to the Ascomycota phylum. Ascomycetes dominate the mycobiome recovered from typical indoor environments, as humans, insects, and other animals contribute significantly to this population [53]. Interestingly, LP1 harbored higher relative abundances of Mortierellomycota compared to other confined environments. Most species of Mortierellomycota are common soil dwellers, frequently associated with plant rhizospheres and endospheres [54, 55]. It is likely that the presence of these organisms is a result of the large plant-growing space in the LP1 system. Moreover, unlike the ISS [16], the LP1 environmental mycobiome resembles that of plant environments (Fig. 6), rather than that of human skin surfaces. Growing plants is therefore critical to the maintenance of diversity, uniqueness, and stability of fungal communities within a BLSS.

Another initial objective of the present study was to identify the effect of crew members and locations on the surface mycobiome. The mycobiome in LP1 was *strongly* influenced *by* different occupants. Group G2 presented significantly greater fungal community diversity when compared with G1 (Fig. 3), indicating that occupant presence is an important determinant of the BLSS mycobiome. A previous study showed that the overall fungal diversity in a closed PCSS habitat changed during human presence [17]. Another study showed that the entry of personnel brought many fungal species to the closed BLSS living environment [56]. Our results confirm these findings and add new insights into how occupant change affects fungal diversity; healthy human individuals impart a significant variation in the taxonomic composition of the mycobiome [57], as evidenced by the different fungal communities between the G1 and G2 groups. Earlier studies did not positively correlate changes in indoor mycobiomes with human presence [50, 58]. These conflicting observations could be associated with whether the building is an open or closed system. A previous study demonstrated that the mycobiome in an open, common indoor space was influenced mainly by airborne fungi [59].

Most strikingly, the effect of occupant change on the fungal community was impacted by location. There was a significant difference in the fungal community on the surface of the equipment in the CC and SC between G1 and G2, while there was no significant difference in the PC. Similarly, our previous study demonstrated that plant cabin air had lower levels of *Penicillium* and *Aspergillus* than occupant living cabin air during a 105-day BLSS analog experiment [56]. Interestingly, all *Aspergillus* and *Penicillium* were assigned to the same fungal traits, i.e., saprotroph and foliar_endophyte, based on their primary and second lifestyles (Table S2). Moreover, the proportion of the pathotroph trophic mode of fungi in the PC was lower than in the other two locations (Fig. S7). These phenomena suggest that the effect of a large plant-growing area on the surface mycobiome in the PC outweighs that of occupant change.

The analysis of mycobiome biomarkers in different locations in LP1 further confirmed this conclusion. *Geomyces* and *Meyerozyma*, as biomarkers to distinguish different locations, showed higher relative abundance in the PC (Fig. 4). Moreover, *Geomyces* and *Meyerozyma* belong to the soil saprotroph and epiphyte, respectively, based on their primary lifestyles (Table S2). Therefore, these two types of fungi should have a close relationship with the plants in the PC. *Geomyces* fungi occur in diverse ecosystems and are abundant throughout growth stages and plant organs [60, 61]. Most *Meyerozyma* fungi play an important role in plant resistance to pathogens [62, 63].

The final objective of our study was the analysis of gene copy numbers associated with mycotoxin biosynthesis. Mycotoxins are secondary metabolites produced by filamentous fungi, including aflatoxins, trichothecenes, fumonisins, and patulin, which are known to include greater than 400 species [63]. Multiple studies have shown that the use of real-time quantitative polymerase chain reaction (qPCR) can enable an accurate analysis of mycotoxin gene expression [25, 26, 64–67]. In these previous studies, the authors developed pairs of *nor1*, *ver1*, *ITS1*, *idh*, and *tri5*-based primers to specifically test for the presence of fungi that produce mycotoxins [68–72].

Regardless of occupant composition and location factors, there was no significant difference in the copy numbers of the *nor1*, *ver1*, *ITS1*, *idh*, and *tri5* toxin genes (Fig. 5 a and c; Figs. S5 & S6). Moreover, the copy numbers of these toxin genes and fungal load (ITS region copy number) did not accumulate over time (Fig. 5b), which may be related to the ecosystem stability of the BLSS. Fungi, including many toxigenic species, are regularly encountered in damp indoor environments [73]. However, LP1 is a controlled environment with constantly controlled temperature and humidity, ensuring system stability. Plant cultivation indoors or in a confined environment does not emit harmful levels of fungal propagules; provided systems are well monitored and maintained [74, 75].

We found a positive correlation between fungal communities and mycotoxin genes (Fig. 5d). Most of the mycotoxin producers can be found in the fungal genera *Aspergillus*, *Penicillium*, *Fusarium*, and *Alternaria*, which concomitantly are the most abundant contaminants of food and feed [25]. However, a positive correlation was apparent between the fungal genera *Cladosporium* and *Apodus* and the mycotoxin-producing genetic markers, *nor1*, *ver1*, and *ITS1*, in our study (Fig. 5). *Nor1*, *ver1*, and *ITS1* have been reported to be markers targeting aflatoxin [71], ochratoxin [69], and fumonisin [70], respectively. *Cladosporium* belongs to the most common molds in indoor and outdoor air, as well as in materials such as soil, plants, textiles, plastics, and foodstuffs [76]. A previous study reported that *Cladosporium* is often associated with *Fusarium*, which is able to produce fumonisin, as a major contaminant of wheat grains [61]. Importantly, wheat was mainly grown and harvested, as the most important food crop, in the Lunar Palace 365 experiment [22]. Furthermore, a recent study showed that the correlation between the *Cladosporium* DNA and the mycotoxin emodin contents was significant in an investigation of contamination of Fabaceae plants [77, 78]. Furthermore, *Cladosporium* fungi require similar growing conditions as *Alternaria*, which is one of the major mycotoxigenic fungal genera. Previous reports have revealed a similarly significant relationship between *Alternaria* and *Cladosporium* fungi coexisting in foods [79] and indoor air [80]. The above explains why *Cladosporium* positively correlated with the copy numbers of toxin genes. *Apodus* are widely distributed in plant rhizospheres and may act as root symbionts to promote plant growth and nutrient supply [81, 82]. As of yet, there has not been a report of mycotoxin secretion from *Apodus*. Therefore, the positive correlation between *Apodus* and toxin genes is somewhat enigmatic. Further research should be undertaken to investigate whether these fungi produce toxins. Nevertheless, *Cladosporium* and *Apodus* may be used as potential biomarkers for monitoring the release of mycotoxins in BLSSs.

From a microbiological point of view, our methods were not perfectly suitable to determine an elevated risk of infection or transfer of mycotoxin-related genes to crew members in a BLSS. However, we consider this risk as extraordinarily low, based on a comparison with a recent investigation on the ISS [83]. Nevertheless, although the risk was deemed low, monitoring microbial dynamics and mycotoxin-related gene expression profiles inside isolated and confined habitats is still very important to understand the impact of isolated events, such as contamination or mycotoxin infection. Importantly, micromycetes producing mycotoxins play a vital role in the so-called sick building syndrome that has recently emerged as a global issue [84]. Studies have shown that fungi may display increased secondary metabolic processes in damp buildings with the potential for greater per cell production of allergens, toxins, and pathogenicity [85–87]. Our work may suggest a potential ecological approach to tackling mycotoxin contamination using green plants in human living settlements.

The present study has limitations and serves only partially as a basis for meaningful recommendations for future attempts to sustain a safe microbial environment in a human outpost on the moon or Mars.

Even though amplicon-based sequencing can be affected by certain biases when using a single ITS1 primer set [88, 89], the mycobiome, obtained with this dataset, was primarily linked to the crew change and location function of the BLSS analog environment. Future fungal community analysis should consider potential primer effects and design the experimental approach accordingly to maximize accuracy. Combining multiple primers targeting the ITS region or a long-read third-generation sequencing approach may be the best strategy to generate a fully accurate view of the fungal composition [90, 91]. While those analogs differ in some important aspects (e.g., gravity and radiation) from spaceflight itself, they place a small, isolated crew in combinations of the following: long-term confinement, high workloads, restricted waste disposal, limited hygiene, and/or low air or water quality. They also offer the possibility to monitor related medical and psychological issues comprehensively. Hence, future missions in preparation for crewed missions to the moon or Mars in the upcoming decades should consider microbial warning systems based on automated sampling technologies, accurate and efficient sequencing analysis, and predictive models comparing expected and true microbial compositions in the habitat and its crew. Nevertheless, our study benefits from its defined confined setup with limited amounts of confounding environmental variables, defined sets of occupants, mass plant growth in a remarkable level of detail, and the correlation of qualitative and quantitative microbial and mycotoxin genes data.

## Conclusion

Our study has shown that the LP1 system, as a BLSS analog, exhibited significant differences in fungal community diversity than other confined habitats, with higher fungal alpha diversity and different community structures. Our study also reveals a diverse and distinctive surface fungal population that changed over crew shifts. Furthermore, plants were the most important sources of surface fungi and had an important effect on maintaining the diversity, uniqueness, and stability of fungal communities within BLSS. There were no significant differences in fungal toxin genes between occupant groups, between time points, or between different locations. Our findings can be used to help develop safe, closed ecosystems that meet the requirements for deep space human habitation. In addition, our results may have a significant impact on our understanding of microbial safety in working and living environments that include plant growth.

## Supplementary Information

**Additional file 1: Supplementary Table S1**. qPCR primer sequences, mycotoxin types, producers, and toxic effects in humans. **Supplementary Table S2**. ASV abundances and potential ecological functions (F01-F70). **Supplementary Table S3**. ISS dust, AIT, ILMAH, and LP1 surfaces merged fungal ASV table. **Supplementary Table S4**. Species abundance tables at the phylum level. **Supplementary Table S5**. Species abundance tables at the genus level. **Supplementary Table S6**. Trophic modes of the fungal microbiome in the different crew groups (G1 and G2) and locations (CC, PC, and SC). **Supplementary Table S7**. Correlation of species at the genus level and mycotoxin genes.

**Additional file 2: Figure S1**. Relative abundance (%) of the major genera present in the fungal microbial communities. **Figure S2**. Comparison of microbial composition in different environments based on SVM. **Figure S3**. Comparison of the diversity of the *Penicillium* between the different crew groups. **Figure S4**. Box and whisker plots of the Shannon diversity index of the HTS dataset according to the day of isolation (time). **Figure S5**. Trophic modes of the fungal microbiome in the different groups (G1 and G2). **Figure S6**. Comparison of community diversity among different occupant groups and different sampling locations. **Figure S7**. Trophic modes of the fungal microbiome in the different locations (CC, PC and SC). **Figure S8**. Comparison of the total expression levels of mycotoxin genes among different occupant groups. **Figure S9**. Comparison of fungal toxin gene expression at different locations. **Figure S10**. Correlation network analysis of the fungal community.

## Data Availability

The dataset of the ITS sequencing data reported in this paper has been deposited in the Genome Sequence Archive (Genomics, Proteomics & Bioinformatics 2017) in the National Genomics Data Center (Nucleic Acids Res 2020), the Beijing Institute of Genomics (China National Center for Bioinformation), and Chinese Academy of Sciences, under accession number CRA003698 that is publicly accessible at https://bigd.big.ac.cn/gsa. All other data are available from the GitHub site (https://github.com/YJL900223/LP_surface_ITS).

## Abbreviations

LP1: Lunar Palace 1
BLSS: Bio-regenerative life support system
ITS1: Internal transcribed spacer region 1
qPCR: Quantitative polymerase chain reaction
ASVs: Amplicon sequence variants
OTU: Operational taxonomic unit
CC: Comprehensive cabin
PC: Plant cabin
SC: Solid waste treatment cabin
ISS: International Space Station
ILMAH: Inflatable lunar/Mars analog habitat
LDA: Linear discriminant analysis
PcoA: Principal coordinates analysis
PERMANOVA: Permutational multivariate analysis of variance
AIT: Assembly, integration, and test
SVM: Support vector machine
PCSS: Physicochemical regenerative life support system
