# Supplementary material for: Surface fungal diversity and several mycotoxin-related genes’ expression profiles during the Lunar Palace 365 experiment
Source: Microbiome. 2022 Oct 12;10:169. doi: 10.1186/s40168-022-01350-8 (PMC9555122; doi:10.1186/s40168-022-01350-8)
Supplement: Supplementary file 3 — Additional file 2: Figure S1. Relative abundance (%) of the major genera present in the fungal microbial communities. Figure S2. Comparison of microbial composition in different environments based on SVM. Figure S3. Comparison of the diversity of the Penicillium between the different crew groups. Figure S4. Box and whisker plots of the Shannon diversity index of the HTS dataset according to the day of isolation (time). Figure S5. Trophic modes of the fungal microbiome in the different groups (G1 and G2). Figure S6. Comparison of community diversity among different occupant groups and different sampling locations. Figure S7. Trophic modes of the fungal microbiome in the different locations (CC, PC and SC). Figure S8. Comparison of the total expression levels of mycotoxin genes among different occupant groups. Figure S9. Comparison of fungal toxin gene expression at different locations. Figure S10. Correlation network analysis of the fungal community. [file 40168_2022_1350_MOESM2_ESM.docx]

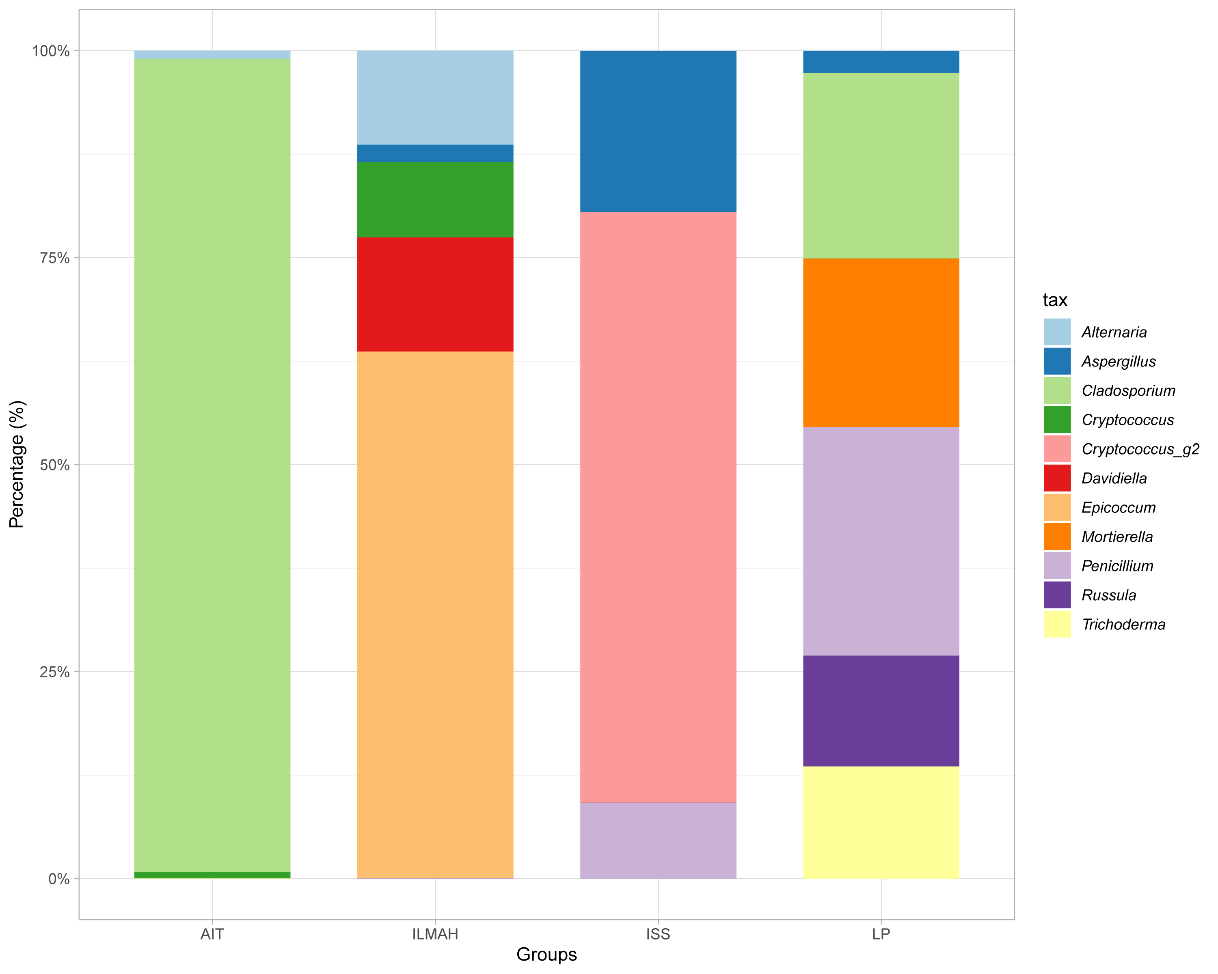


**Figure S1. Relative abundance (%) of the major genera present in the fungal microbial communities.** LP: Lunar Palace 1; ISS: International Space Station; AIT: assembly, integration, and test center; ILMAH: Inflatable Lunar/Mars Analog Habitat


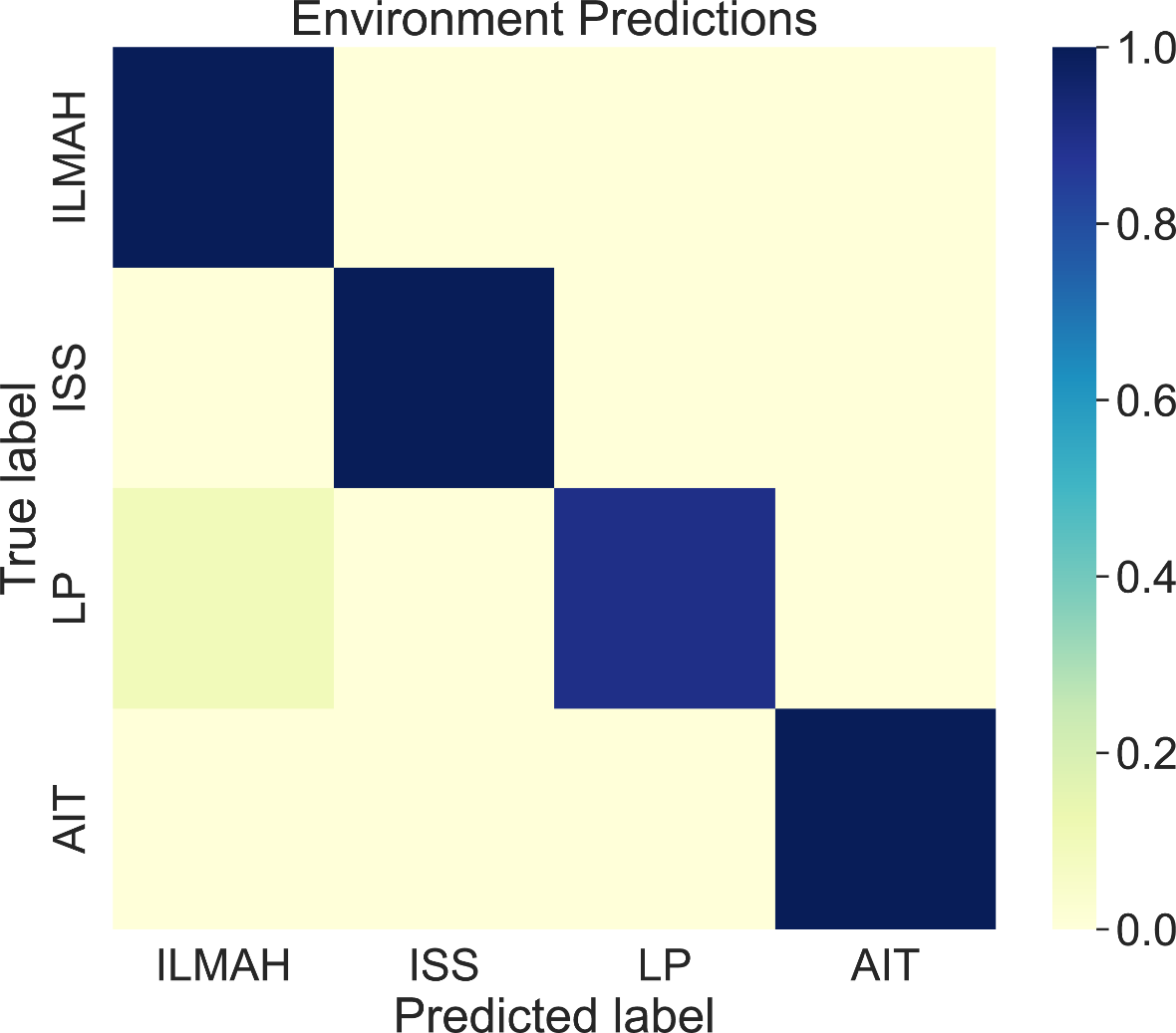


**Figure S2.** **Comparison of microbial composition in different environments based on SVM.** LP: Lunar Palace 1; ISS: International Space Station; AIT: assembly, integration and test center; ILMAH: Inflatable Lunar/Mars Analog Habitat


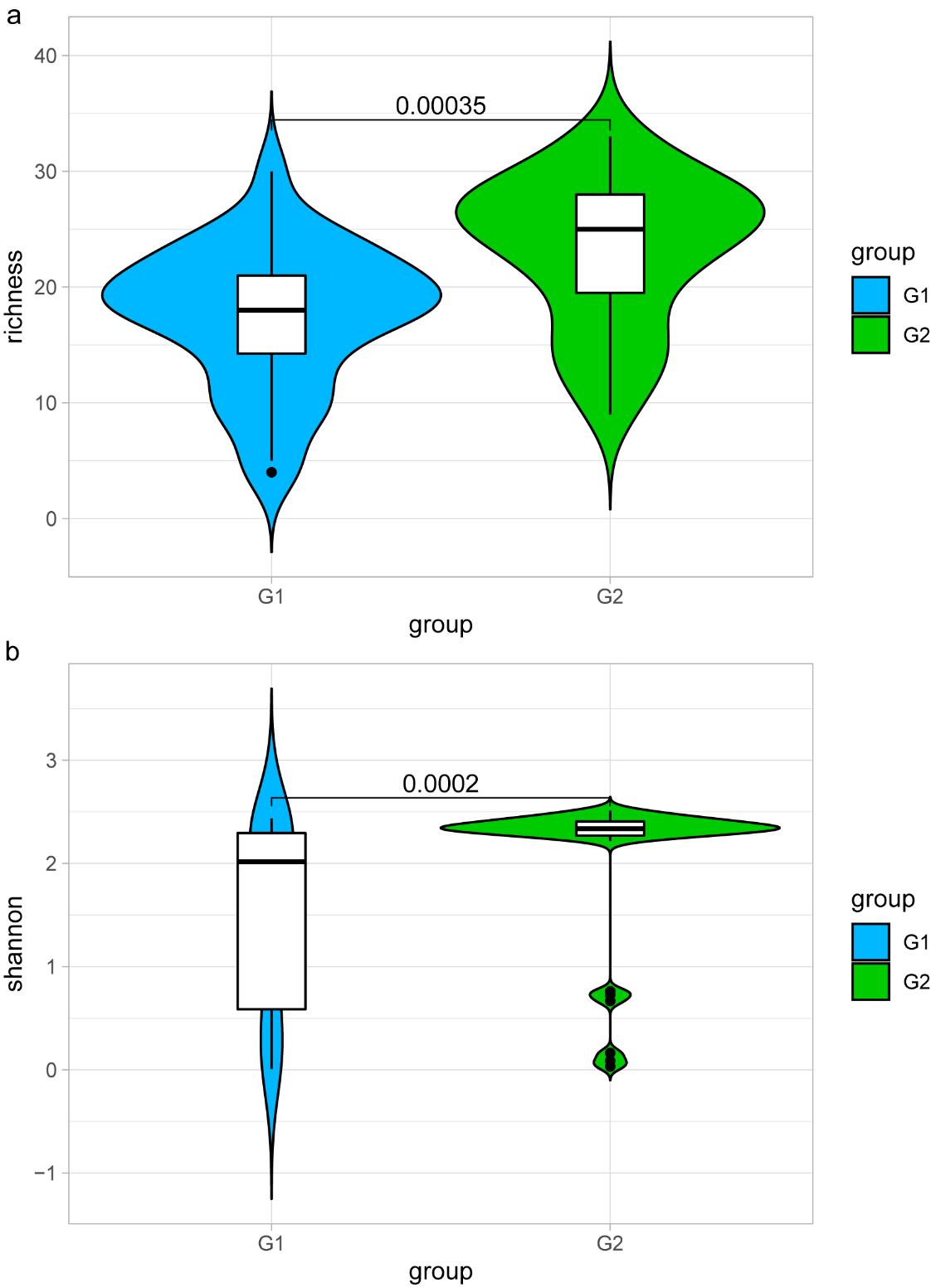


**Figure S3. Comparison of the diversity of the *Penicillium* between the different crew groups**. a. Comparison of *Penicillium*’s species richness between the different occupant groups at the richness index. b. The Shannon diversity of *Penicillium* among the different occupant groups


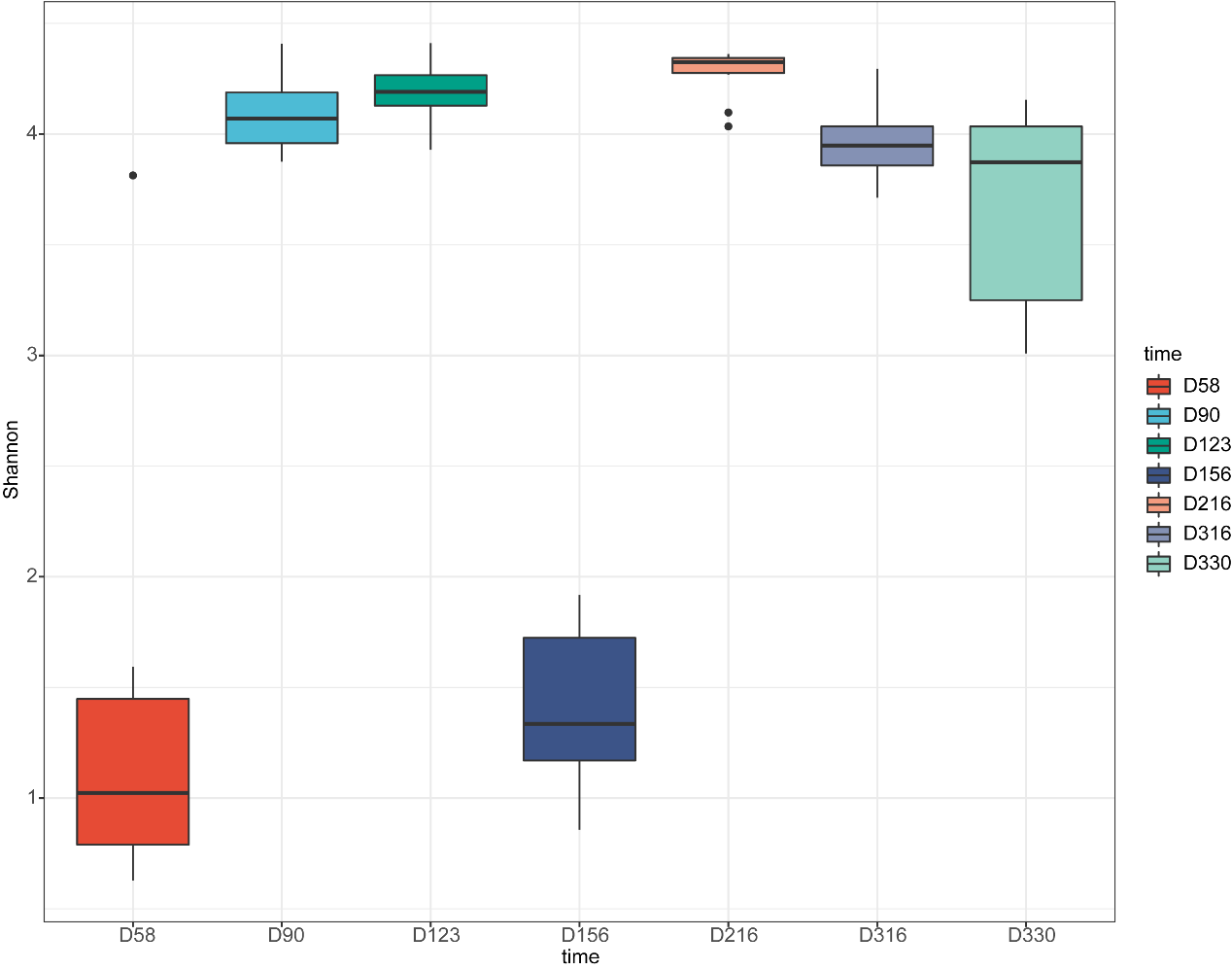


**Figure S4. Box and whisker plots of the Shannon diversity index of the HTS dataset according to the day of isolation (time).**


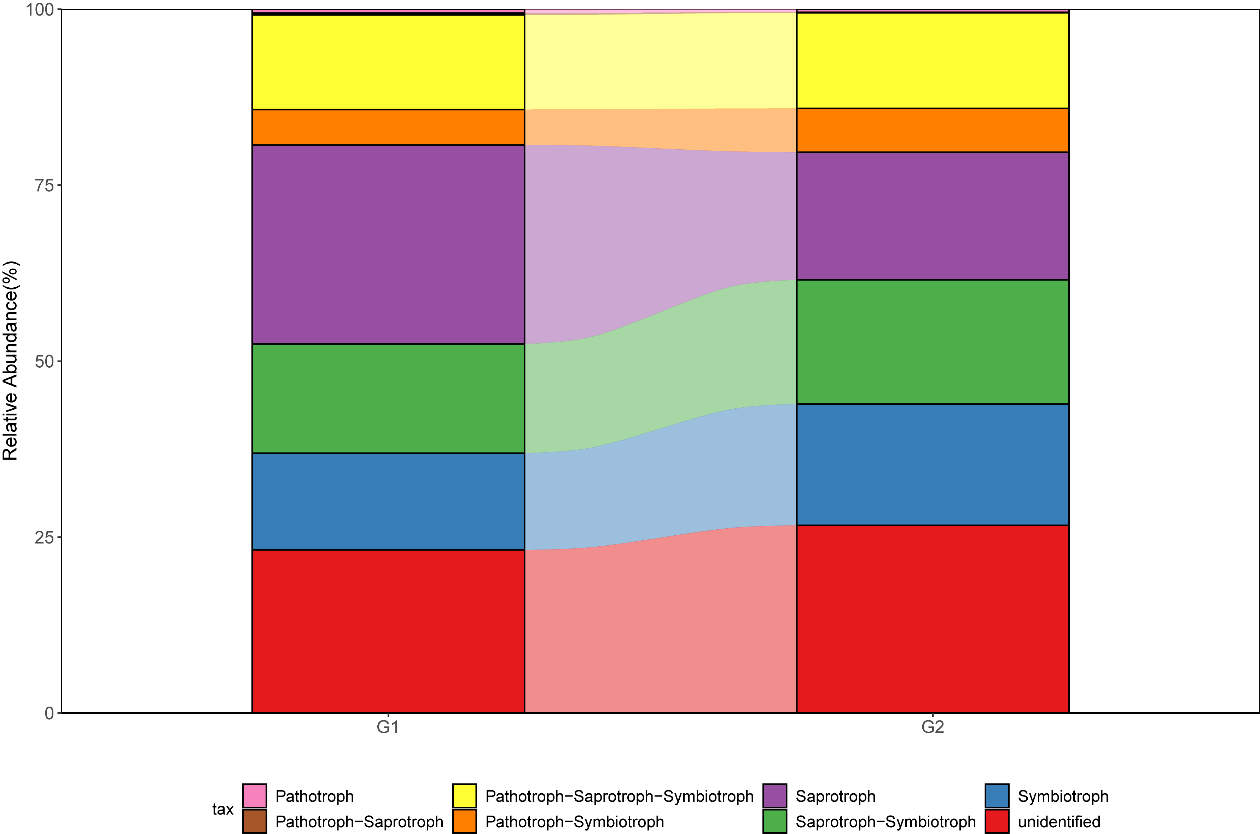


**Figure S5. Trophic modes of the fungal microbiome in the different groups (G1 and G2).**


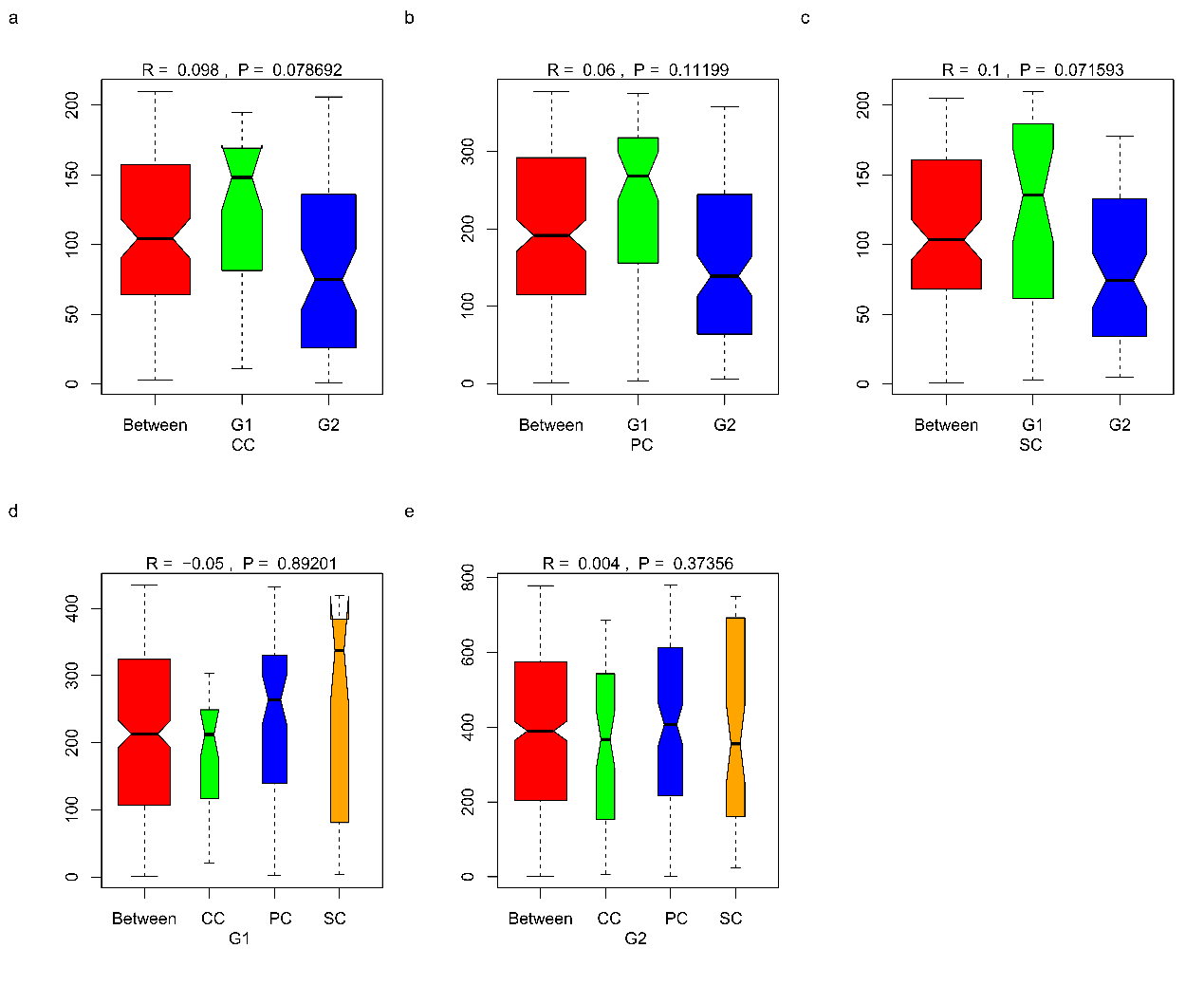


**Figure S6. Comparison of community diversity among the different occupant groups and different sampling locations**. a. Comparison of community diversity at the CC location among the different occupant groups. b. Comparison of community diversity at the PC location among the different occupant groups. c. Comparison of community diversity at the SC location among the different occupant groups. d. Comparison of community diversity among the different sampling sites for the G1 occupant group. e. Comparison of community diversity among different sampling sites for the G2 occupant group. CC, comprehensive cabin; PC, plant cabin; SC, solid waste treatment cabin.


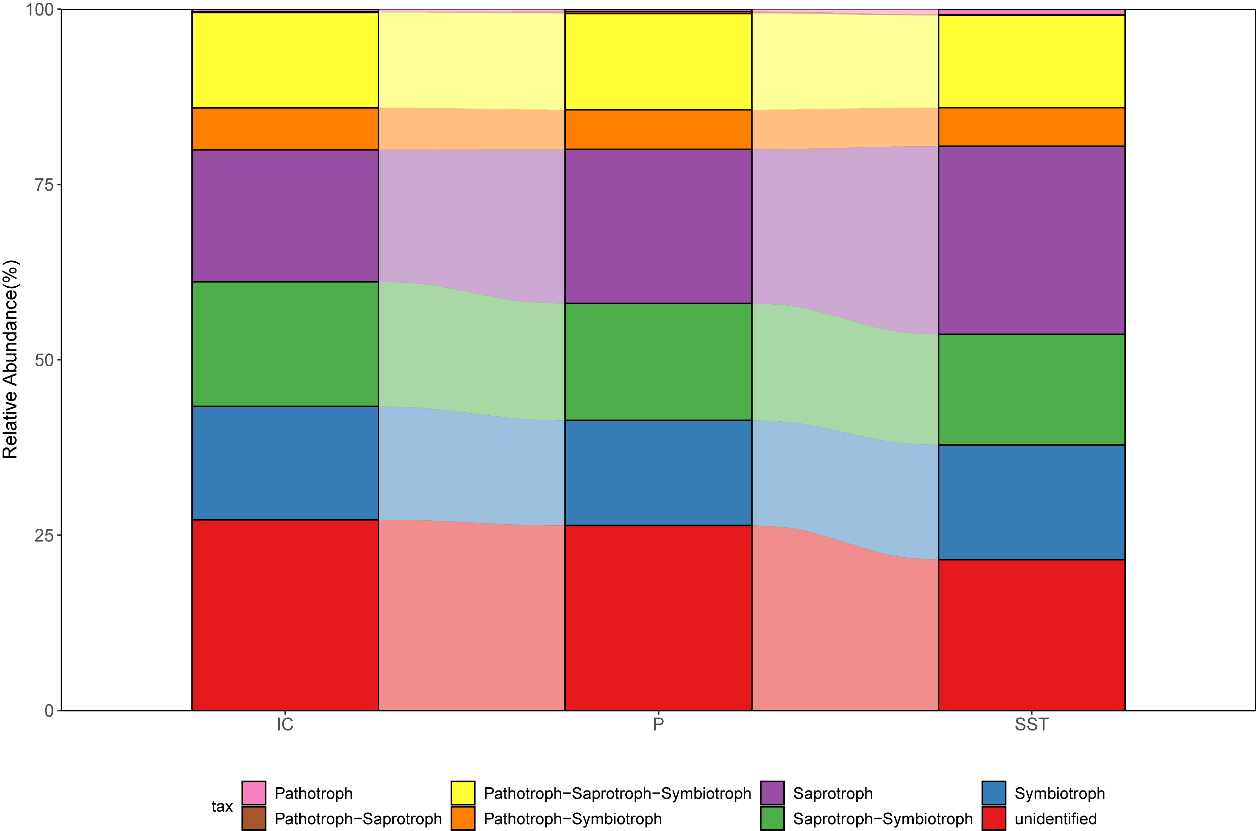


**Figure S7. Trophic modes of the fungal microbiome in the different locations (CC, PC and SC).**


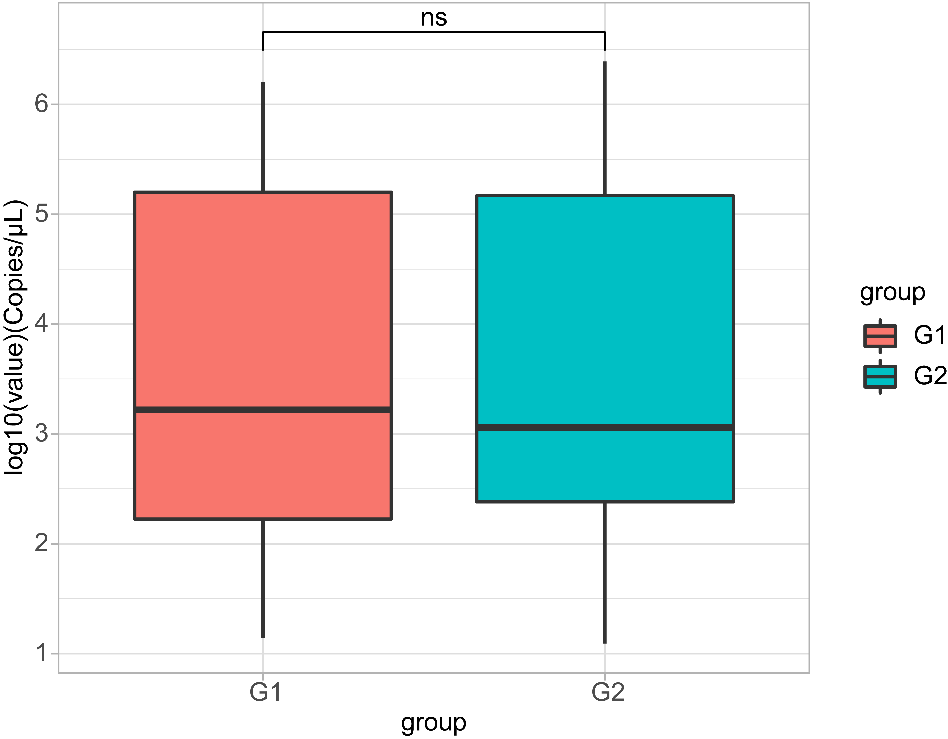


**Figure S8. Comparison of the total expression levels of mycotoxin genes among different occupant groups.** *p* value > 0.05 is indicated by "ns" for not significant.


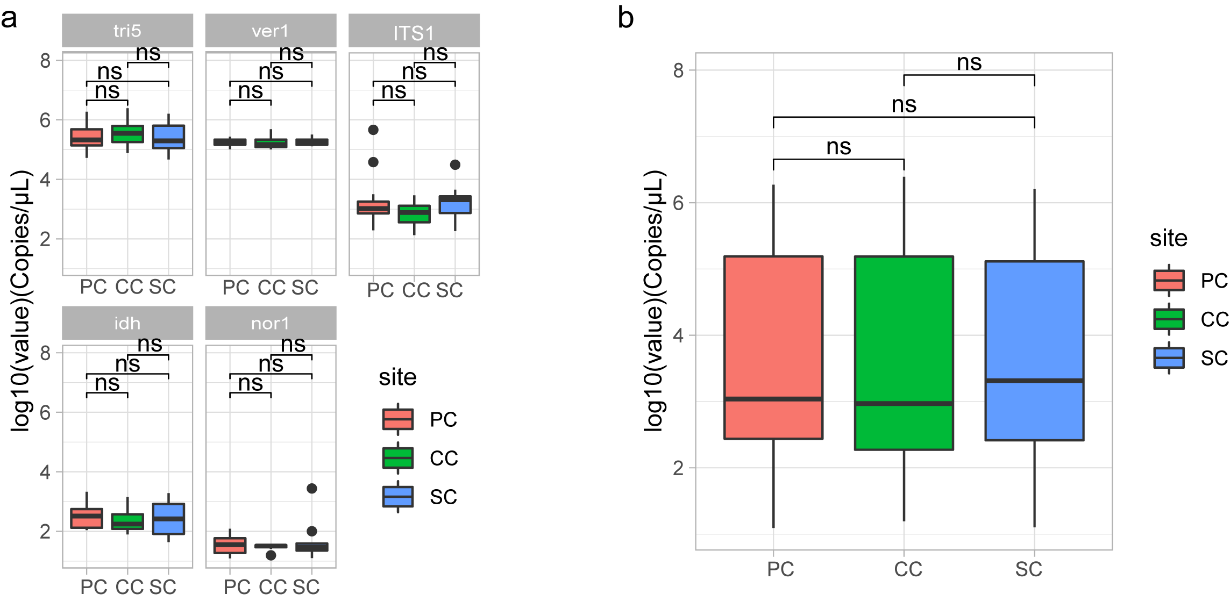


**Figure S9. Comparison of fungal toxin gene expression at the different locations.** a. Comparison of the expression of each mycotoxin gene between the different locations. b. Comparison of the total expression levels of mycotoxin genes between the different locations.


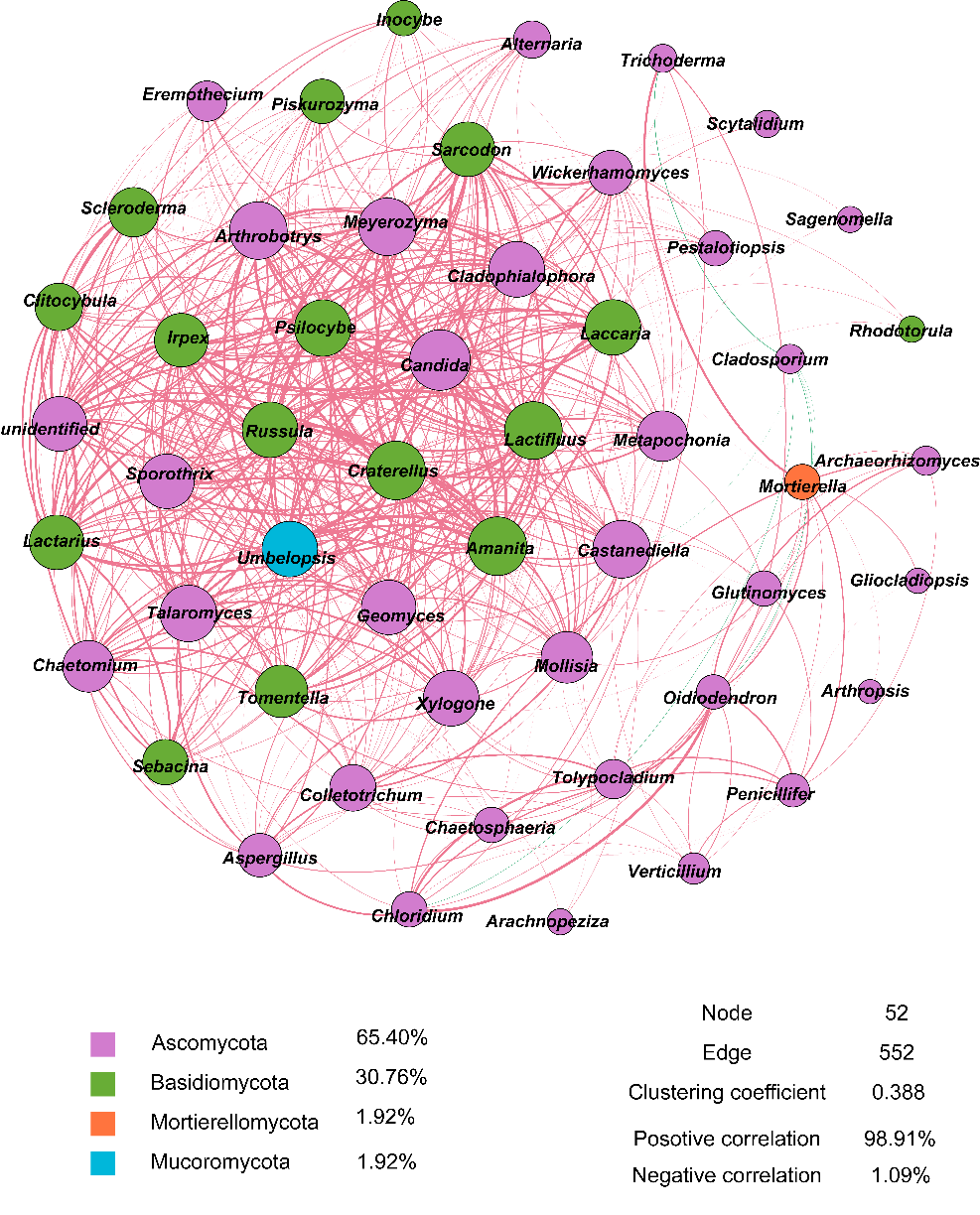


**Figure S10. Correlation network analysis of the fungal community**. Species from different phyla are represented by nodes in the network diagram. Fungi at different phylum levels are distinguished by color. The size of each node corresponds to the quantity of the corresponding Fungi. The connection between any two nodes indicates two genera with a markedly high correlation (Spearman’s |r| > 0.7; *p* < 0.01). Red line, positive correlation; green line, negative correlation
